# Physiological response to a breed evaluation field test in Icelandic horses

**DOI:** 10.1017/S1751731113002309

**Published:** 2014-01-06

**Authors:** G. J. Stefánsdóttir, S. Ragnarsson, V. Gunnarsson, A. Jansson

**Affiliations:** 1 Department of Equine Science, Hólar University College, 551 Sauðárkrókur, Iceland; 2 Department of Animal Nutrition and Management, Swedish University of Agricultural Sciences, P.O. Box 7024, 750 07 Uppsala, Sweden

**Keywords:** exercise physiology, hematological parameters, lactate, heart rate, Icelandic horse

## Abstract

This study examined the response in terms of heart rate (HR), respiratory rate (RR), haematocrit (Htc), rectal temperature (RT), and some plasma variables in Icelandic horses of different sexes and ages performing the riding assessment in a breed evaluation field test (BEFT). The study was conducted in Iceland on 266 horses (180 mares and 86 stallions, divided into four age groups; 4, 5, 6 and ⩾7 years old). RT and RR were recorded and blood samples were taken before the warm-up and after the riding assessment. Horse HR, velocity and distance were recorded during the warm-up, the riding assessment and a 5-min recovery period. The distance covered in the BEFT was 2.9±0.4 km (range: 1.8 to 3.8 km, *n*=248), the duration was 9:37±1:22 min:s (range: 5:07 to 15:32 min:s, *n*=260) and the average speed was 17.8±1.4 km/h (range: 13.2 to 21.3 km/h, *n*=248). Average HR was 184±13 b.p.m. (range: 138 to 210 b.p.m., *n*=102) and peak HR 224±9 b.p.m. (range: 195 to 238 b.p.m., *n*=102), and 36% of the BEFT was performed at HR ⩾200 b.p.m. Post-exercise plasma lactate concentration (Lac) was 18.0±6.5 mmol/l (range: 2.1 to 34.4 mmol/l, *n*=266), and there was an increase in total plasma protein, plasma creatine kinase and aspartate amino transferase concentration, as well as RR, RT and Htc. Stallions covered a longer total distance (in the warm-up and BEFT) (*P*<0.05), at a faster speed during BEFT (*P*<0.001) than mares and had higher Htc and lower HR and post-exercise Lac values. There were few effects of age, but the 4- and 5-year-old horses had lower Htc than older horses and 4-year-old horses had higher post-exercise RR than older horses, although they were ridden for a shorter distance, shorter duration and at lower peak velocity (*P*<0.1). The results showed that the riding assessment in the BEFT is a high-intensity exercise. The results also showed that aerobic fitness was higher in stallions and that age had a limited effect on the physiological response. It is suggested that these results should be used as a guide for the development of training programmes and fitness tests in Icelandic horses that would improve both performance and welfare of the horse.

## Implications

The Icelandic horse breed is widespread and its popularity is increasing as a leisure and sport horse for gait competitions. However, knowledge is lacking on the physiological response of Icelandic horses to exercise and competitions. Therefore, it is an animal welfare issue to generate such knowledge, so that training programmes and competition content can be adjusted to support the general welfare of the breed.

## Introduction

The Icelandic horse is a purebred gaited riding horse originating from Iceland (International Federation of Icelandic Horse Associations (FEIF), 2002), but located in 33 countries outside Iceland and bred in 18 countries (WorldFengur, 2013). The breed evaluation system and the studbook WorldFengur (2013) are also international (FEIF, 2002). The official breeding goal includes 15 traits (eight conformation and seven riding ability traits) that are assessed individually at breeding shows, hereafter referred to as the breed evaluation field test (BEFT), which is standard for all countries (FEIF, 2002). In recent years (2008 to 2012), 1700 to 2800 horses attended a BEFT every year, of which 1000 to 1800 were in Iceland (FEIF, 2013). For comparison, the number of live foals registered in FEIF countries annually in 2008 to 2012 ranged from 10 559 to 14 866, of which 4861 to 7399 were in Iceland (WorldFengur, 2013).

The BEFT consists of three parts: (1) objective body measurements, (2) judging of conformational traits and (3) riding abilities. Scores for conformation and riding abilities are weighed to give the final total score. Riding abilities (performance in walk, tölt, trot, flying pace (pace) and canter/gallop, as well as spirit and general impression) account for 60% of the total score and conformation for 40% (FEIF, 2002). It is known that stallions obtain higher total scores than mares and that total score increases with age from 4 to 6 years and older (Árnason, 1984). This implies that fitness and also physiological characteristics related to age and sex could be important for performance. It is known from other breeds that performance is higher in stallions than in mares (Buttram *et al.*, 1988; Persson, 1997) and increases with age, at least to a certain limit (Seeherman and Morris, 1991) and that sex, age (Ronéus *et al.*, 1991; Ronéus, 1993) and training (Ronéus *et al.*, 1992) influence, for example, muscle fibre composition and also blood volume and haematocrit (Htc) (Persson *et al.*, 1996).

To the best of our knowledge, no published data are available on the physiological response of Icelandic horses to a BEFT. In fact, the physiological responses of this breed to exercise have not been assessed, except in a few pilot studies (unpublished results). However, speed observations from competitions (WorldFengur, 2013) and heart rate (HR) and blood lactate responses from the pilot studies indicate that Icelandic horses are subjected to high workloads. Therefore, the aim of this study was to describe the response in terms of heart and respiratory rate (RR), Htc, rectal temperature (RT), and some plasma variables in stallions and mares of different ages performing a BEFT. Our starting hypotheses were that the BEFT is a high-intensity exercise and that some physiological responses could be affected by sex and age.

## Material and methods

The study was approved by the National Animal Research Committee of Iceland. The study was conducted at BEFT in Hella in southern Iceland on 30 May to 2 June and 6 to 9 June 2011. Owners and trainers were informed that participation in the study was voluntary.

### BEFT and design of the study

The riding abilities of a horse are judged in two separate assessments. In the first and main assessment, the horse is expected to show all its gaits, but exactly how this is done is decided by the rider. The horse is ridden alone on a straight field track (250 to 300 m long, 4 to 6 m wide) and 200 m of the track is used for judging and 50 m at each end to turn around. The horse has to be ridden along the track a minimum of six times and a maximum of 10 times, that is, three to five times in each direction (FEIF, 2002). The second assessment is voluntary and is carried out if the rider/trainer/owner would like to try to improve the scores from the first assessment. In the present study, only the results from the first assessment were included.

A horse must be at least 4 years old in the calendar year to be judged for riding abilities in BEFT. Stallions and mares are shown in separate sex and age classes (4, 5, 6 and ⩾7 years old) and geldings are shown in one group irrespective of age (FEIF, 2002), as very few are judged annually (WorldFengur, 2013). Only mares and stallions were included in the present study. According to the judging scale used in a BEFT, each trait is scored within the range 5.0 to 10.0, with 0.5 increments. A score of 5.0 is given if a gait is not shown and 10.0 is given for an excellent trait (FEIF, 2002). Three internationally accepted judges generally work in a committee and give a joint score for each trait (FEIF, 2002). During the present study six judges worked, three at a time. Immediately after a horse has finished the riding assessment, there is also an obligatory shoe and health check (legs and mouth), which lasts for about 1 to 2 min.

### Horses

In total, 396 horses were shown at the BEFT and the study included 266 of these, 86 stallions and 180 mares, leaving 130 in a ‘non-study’ group. The horses were mainly fed roughage 5.7±1.5 kg (range: 0 to 12 kg, *n*=254) and some (at least 10%) were also grazed (0.5 to 6.0 h/day), whereas one horse was grazed only. Most horses (68%) were given some concentrates (1.1±0.5 kg, range: 0.2 to 4 kg, *n*=182). The inclusion criteria were that the owners/trainers were willing to take part, and that the horses were judged for both conformation and riding abilities and had completed both parts of the BEFT. One of the horses in the study was later disqualified and did not get a score because of deviation from the rules of legal shoes.

### Data collection

Before the riding assessment, the BW of each horse and rider and the weight of a spot sample of saddles (23 saddles and blankets) were recorded using an electronic livestock scale (Smartscale 300; Gallagher, Hamilton, New Zealand). The ratio of rider BW (riding tack not included) to horse BW was calculated (BWR) and used as a variable in the statistical analyses. The riders answered a questionnaire about the horse regarding name, age, feeding, travel time to the showground and the level of preparation for the BEFT (scale 1 to 10, 1=badly and 10=very well prepared). The horses were given a body condition score (BCS) according to the specific BCS-scale devised for Icelandic horses (scale 1 to 5; Stefánsdóttir and Björnsdóttir, 2001).

RT (digital thermometer; Disney, Hartmann, Heidenheim, Germany) was measured and RR was recorded by counting breaths both before the horses were warmed up before the riding assessment and within 5 min after the riding assessment. Blood samples were also taken from the jugular vein by Vacutainer in chilled lithium heparinised tubes (9 ml, Vacuette^®^; Greine-Bio-One, Kremsmuenster, Austria) before the warm-up and the riding assessment and within 5 min after the horse left the track after performing the riding assessment. The same person recorded RT and RR and collected the blood samples.

During the warm-up and riding assessment, and for a 5-min recovery period after the assessment HR (Polar HR Monitor RS800CX and belt with Polar Equine T56H transmitter W.I.N.D., Kempele, Finland), velocity and distance covered (Polar G3 GPS sensor, Kempele, Finland) were recorded. According to the manufacturer, the accuracy of distance measurements is ±2% and that of velocity measurements is ±2 km/h. The HR monitors were set to record in 1 s mode. All horses were videotaped during the riding assessment, using a digital HD video-camera recorder (Sony HDR CX360VE, Tokyo, Japan). All watches used in the study (on HR monitors, the video camera and watches used to record times) were synchronised and the video recordings were used to check and synchronise the start and end time of the riding assessment. The riding track was labelled with bars every 50 m for 300 m. Scores from the riding assessment and information on wither height (WH) were obtained from the official studbook (WorldFengur, 2013).

The weather at the showground was recorded every 5 min (two to four observations per horse) by an automatic weather station (Art. no. 36-3242, Model WH-1080; Clas Ohlson, Insjön, Sweden). The ambient temperature at the track was 10.7±2.0°C (range: 9.0 to 14.1°C), the relative humidity 38±14% (range: 24% to 60%), the wind speed 5.6±2.3 m/s (range: 3.5 to 9.8 m/s) and average strongest wind speed 7.6±2.9 m/s (range: 5.0 to 12.9 m/s).

### Blood analysis

Blood samples were kept chilled and Htc and haemoglobin (Hb) were analysed within 30 min. Htc was analysed using non-heparinised capillary tubes, centrifuged at 18 840×**g** (Sigma 1–15; Laborzentrifugen GmbH, Osterode am Harz, Germany) for 6 min. Triple measurements were performed and a mean value was used for the statistical analyses. Hb concentration was analysed using a Hemocue POC analyser (Helsingborg, Sweden). Plasma was separated by centrifugation (15 min, 520×**g**, Hettich, Tuttlingen, Germany) and stored at −18°C until analysis of lactate, creatine kinase (CK), aspartate amino transferase (AST) and total plasma protein concentration (TPP). Plasma lactate concentration (Lac) was analysed after ~4 months using an enzymatic (l-lactate dehydrogenase and glutamate-pyruvate transaminase) and spectrophotometric method (Boehringer Mannheim/R-Biopharm, Darmstadt, Germany) with a CV of 2.3% according to the manufacturer. All duplicates showing variation >10% were re-analysed until the variation was <10%. The CV of the plasma lactate analyses was 3.3%. CK and AST were analysed after ~4 months using an enzymatic method (spectrophotometer, Architect c4000, Abbott Park, IL, USA). TPP was analysed after ~12 months using a refractometer (Atago, Tokyo, Japan).

### Data handling

All HR recordings were transported using an infrared USB Adapter (IrDA, Kempele, Finland) to the software Polar Pro Trainer 5 Equine Edition (Kempele, Finland) and then transferred to Microsoft Excel 2010 for further analysis. Only HR recordings where a minimum of 80% of the recording for the whole riding assessment was considered reliable were used, which resulted in 102 observations. On average, 6±6% (34±34 s/horse) of the recordings from the 102 horses were excluded from the analysis.

### Statistical analysis

Means and standard deviations were calculated for descriptive data and Pearson’s correlations were performed in SAS (Statistical Analysis Systems package 9.2, Cary NC, USA). ANOVA was performed using Proc GLM (model 1 and 2) and Proc MIXED (model 3 and 4) in SAS. The results are expressed as least square means (LS Means) with their root mean square error (RMSE). For comparison, the Tukey test was used and level of statistical significance was set to *P*<0.05.

The BCS, WH, BW and level of preparation were analysed using model (1): *Y*_*ij*_=*µ*+*α*_*i*_+*β*_*j*_+*e*_*ij*_, where *Y*_*ij*_ is the observation/parameter, *µ* the mean value, *α*_*i*_ the fixed effect of sex, *β*_*j*_ the fixed effect of age group and *e*_*ij*_ the residuals; *e*_*ij*_∼(0, *δ* ^2^).

The before and after samples and whether the two groups of horses (study and non-study) differed in total score for riding abilities were analysed using model (2): *Y*_*ijk*_=*µ*+*α*_*i*_+*β*_*j*_+*ε*_*k*_+*e*_*ijk*_, where *Y*_*ijk*_ is the observation/parameter, *µ* the mean value, *α*_*i*_ the fixed effect of sex, *β*_*j*_ the fixed effect of age group, *ε*_*k*_ the fixed effect of sample/group of horse and *e*_*ijk*_ the residuals; *e*_*ijk*_∼(0, *δ* ^2^).

The variables distance, speed and duration of warm-up, and the BEFT were analysed using model (3): *Y*_*ijkr*_=*µ*+*α*_*i*_+*β*_*j*_+*ε*_*k*_+*a*_*r*_+*e*_*ijkr*_, where *Y*_*ijkr*_ is the observation/parameter, *µ* the mean value, *α*_*i*_ the fixed effect of sex, *β*_*j*_ the fixed effect of age group, *ε*_*k*_ the fixed effect of whether a horse was scored for pace or not, *a*_*r*_ the random effect of the rider and *e*_*ijkr*_ the residuals; *e*_*ijkr*_∼(0, *δ* ^2^). Interactions between fixed factors were tested in the model, but no effect was found for any of the variables tested. The random factor ‘rider’ accounted for 5% to 46% of the random variation in the models for distance, duration and speed, that is, the variation that was not explained by the fixed factors.

The physiological responses (overall effect) to the BEFT and the total score for riding abilities were analysed using model (4): *Y*_*ijklmnr*_=*µ*+*α*_*i*_+*β*_*j*_+*ε*_*k*_+*γ*_*l*_+*η*_*m*_+*τ*_*n*_+*a*_*r*_+*e*_*ijklmnr*_, where *Y*_*ijklmnr*_ is the observation, *µ* the mean value, *α*_*i*_ the fixed effect of sex, *β*_*j*_ the fixed effect of age group, *ε*_*k*_ the fixed effect of whether a horse was scored for pace or not, *γ*_*l*_ the average velocity of a horse in the BEFT as a continuous variable, *η*_*m*_ the distance ridden in the BEFT as a continuous variable, *τ*_*n*_ the BWR as a continuous variable, *a*_*r*_ the random effect of the rider and *e*_*ijklmr*_ the residuals; *e*_*ijklmnr*_∼(0, *δ* ^2^). The random factor ‘rider’ accounted for 0% to 18% of the random variation in the models for physiological responses to the BEFT and for total score for riding abilities, that is, the variation that was not described by the fixed factors.

## Results

### Description of horses, riders and riding tack included in the study

The mean travel time to the BEFT was 31±31 min (range: 3 to 180 min) but two horses had travelled for 6 h on the previous day.

The scores for riding abilities of the horses participating in the study (*n*=265) and the non-study horses (*n*=130) were not different (*P*>0.05) for the whole group (7.69 *v*. 7.72, RMSE=0.32). In addition, there were no differences between the age groups within the studied group and non-studied group (4-year-olds: 7.52 *v*. 7.41, RMSE=0.29, 5-year-olds: 7.71 *v*. 7.71, RMSE=0.27, 6-year-olds: 7.79 *v*. 7.89, RMSE=0.33, ⩾7-year-olds: 7.79 *v*. 7.85, RMSE=0.36) or the sex groups (stallions: 7.82 *v*. 7.79, RMSE=0.34; mares: 7.59 *v*. 7.66, RMSE=0.30). The average age of the horses was 5.9±1.4 year (range: 4 to 11 years, *n*=266; Table 1). The WH was 141.0±2.7 cm (range: 134.0 to 149.0 cm, *n*=266), and was greater in stallions than in mares (141.9 *v*. 140.4 cm, RMSE=2.6, *P*<0.001), but did not differ (*P*>0.05) between age groups. The BW was 339±19 kg (range: 289 to 397 kg, *n*=264) and was not affected by sex (stallions: 340 *v*. mares: 338 kg, RMSE=19, *P*>0.05). However, 4- and 5-year-old horses were lighter than horses ⩾7 years old (333, 335 and 346 kg, respectively, RMSE=19, *P*<0.01), whereas the BW of 6-year-olds (342 kg, *P*>0.05) did not differ from that of the other age groups. The BCS of stallions was lower than that of mares (2.9 *v*. 3.1, RMSE=0.3, *P*<0.001) and did not differ (*P*>0.05) between age groups.

**Table 1:** Number of horses participating in the study, divided into sex and age group

	Age group				
	4 years	5 years	6 years	7 years and older	Total
Mares	19	55	55	51	180
Stallions	18	28	19	21	86
Total	37	83	74	72	266

The study involved 69 riders, who each rode 1 to 29 horses. Five riders rode ⩾10 horses and 13 riders ⩾5 horses, whereas 56 riders rode 1 to 4 horses. Rider BW was 83±11 kg (range: 59 to 112 kg, *n*=67) and weight of saddle and blanket was 8.7±1.0 kg (range: 6.8–10.4 kg, *n*=23). According to the riders, there was a wide range in the level of preparation (2% scores 1.0 to 3.9, 36% scores 4.0 to 6.9 and 62% scores 7.0 to 10.0, *n*=248).

### Duration, distance, and speed of the warm-up and BEFT

Duration, distance, and speed of the warm-up were not affected (*P*>0.05) by sex, age group or whether the horse showed pace or not in the BEFT (Table 2). The distance of the BEFT was 2.9±0.4 km (range: 1.8 to 3.8 km, *n*=248), the duration was 9:37±1:22 min:s (range: 5:07 to 15:32 min:s, *n*=260) and the average speed during the BEFT was 17.8±1.4 km/h (range: 13.2 to 21.3 km/h, *n*=248). Most horses (41%) were ridden ⩾3.0 km, whereas 20% were ridden <2.5 km and 39% were ridden 2.5 to <3.0 km.

**Table 2:** Duration, distance, and speed of stallions and mares in warm-up and the breed evaluation field test (BEFT)^1^ and effects of sex, age group (age: 4, 5, 6 and ⩾7 years) and whether flying pace (Pace) was shown or not

	Stallions				Mares					*P*-values		
	*n*	Mean	Min	Max	*n*	Mean	Min	Max	RMSE	Sex	Age	Pace
Warm-up												
Distance (km)	85	2.2	0.3	4.7	164	2.0	0.2	6.3	0.8	0.070	0.542	0.654
Duration (min:s)	85	19:06	03:05	49:02	174	18:48	02:00	50:28	08:47	0.806	0.786	0.088
Average speed in warm-up (km/h)^2^	85	7.2	2.3	16.8	164	6.9	2.0	13.2	2.3	0.356	0.838	0.175
Average speed, moving in warm-up (km/h)^3^	85	10.8	5.7	19.7	164	10.8	5.8	17.0	2.1	0.886	0.949	0.459
Peak speed in warm-up	85	23.5	14.9	38.5	164	23.0	15.4	38.7	2.9	0.217	0.440	0.934
BEFT												
Distance (km)	85	2.9	1.8	3.6	162	2.8	1.8	3.8	0.3	0.027	0.064	0.013
Duration (min:s)	85	09:40	05:17	15:32	174	09:33	06:10	13:27	01:07	0.472	0.050	0.717
Average speed (km/h)	85	18.0	13.2	21.1	162	17.4	13.4	21.3	1.1	0.001	0.212	0.003
Peak speed (km/h)	85	42.1	32.0	56.8	162	42.0	33.5	52.2	3.3	0.826	0.068	0.718
Total for warm-up and BEFT												
Distance (km)	85	5.1	3.5	7.3	162	4.8	2.9	9.4	0.8	0.014	0.288	0.537
Duration (min:s)	85	28:36	12:04	60:07	174	28:13	11:24	60:40	08:50	0.752	0.700	0.084

1 Values presented as least square means (mean) and root mean standard error (RMSE) and minimum (min) and maximum (max) values.

2 The average speed in the complete duration of warm-up.

3 The average speed in the duration of warm-up after excluding the part when horses and riders were standing still and waiting to start the riding assessment.

Stallions covered a longer distance in the BEFT than mares (*P*<0.05) and horses that showed pace (score ⩾5.5 for pace) covered a longer distance than those that did not (score=5.0 for pace) (2.9 *v*. 2.8 km, RMSE=0.3, *P*<0.05; Table 2). Stallions were ridden at a faster speed than mares (*P*<0.001) and horses awarded a score for pace were faster than horses that received no score for pace (18.0 *v*. 17.5 km/h, RMSE=1.1, *P*<0.01; Table 2). Stallions covered a longer distance in total (in warm-up and BEFT) than mares (*P*<0.05; Table 2).

There was a tendency for 4-year-old horses to be ridden a shorter distance in the BEFT than 5-, 6- and ⩾7-year-olds (2.7 *v*. 2.8, 2.9, 2.9 km, RMSE=0.3, *P*=0.06), for a shorter duration (9:12 *v*. 9:35, 9:48 and 9:53 min:s, RMSE=1.06, *P*=0.05) and at lower peak velocity (40.7 *v*. 42.5, 42.6, 42.5 km/h, RMSE=3.3, *P*=0.07).

### Physiological responses to the BEFT

Measured RR, RT, Htc, Hb, CK, AST and TPP increased following the BEFT (*P*<0.001; Table 3). The HR during the BEFT was 184±13 b.p.m. (range: 138 to 210 b.p.m., *n*=102) and during 36% of the BEFT HR was ⩾200 b.p.m., for 28% it was 180 to 199 b.p.m., for 15% it was 161 to 179 b.p.m. and for 21% it was <160 b.p.m. Peak HR during the BEFT was 224±9 b.p.m. (range: 195 to 238 b.p.m., *n*=102). Mean HR as a proportion of peak HR during the BEFT was 82% (range: 64% to 90%). The HR during the BEFT was affected by velocity (*P*<0.001) and increased by 4 b.p.m. per km/h. Wind speed was positively correlated with the time when HR was ⩾200 b.p.m. in the BEFT (*r*=0.25, *P*<0.05) The strongest wind speed was positively correlated with mean HR in the BEFT (*r*=0.23, *P*<0.05) and with the time when HR was ⩾200 b.p.m. in the BEFT (*r*=0.27, *P*<0.01).

**Table 3:** Physiological responses in stallions and mares before warm-up and after the breed evaluation field test (BEFT)^1^ and effects of sex, age group (age; 4, 5, 6 and ⩾7 years), whether flying pace (Pace) was shown or not, distance (Dist.), velocity (Vel.) and BWR^2^

	Stallions				Mares					*P*-values					
	*n*	Mean	Min	Max	*n*	Mean	Min	Max	RMSE	Sex	Age	Pace	Dist.	Vel.	BWR
RR before (breaths/min)	84	29	16	72	158	30	12	76	11	0.983	0.904	0.548	0.236	0.311	0.972
RR after (breaths/min)	84	103	40	160	159	103	12	168	28	0.915	0.015	0.363	0.415	0.001	0.398
RT before (°C)	84	37.8	36.7	38.5	158	37.8	36.9	38.6	0.3	0.770	0.419	0.979	0.527	0.546	0.414
RT after (°C)	84	39.4	38.0	40.9	158	39.6	38.4	41.1	0.5	0.041	0.796	0.566	0.015	0.105	0.410
RT change (°C)	84	1.7	0.4	3.4	158	1.8	0.5	3.7	0.5	0.067	0.510	0.512	0.029	0.060	0.144
Haematocrit before (%)	84	39	27	50	159	34	25	42	3	<0.001	0.189	0.327	0.830	0.452	0.086
Haematocrit after (%)	84	49	41	55	159	43	36	50	2	<0.001	<0.001	0.275	0.934	0.933	0.085
Hb concentrationbefore (g/l)	83	140	110	197	149	122	95	151	13	<0.001	0.243	0.839	0.834	0.181	0.030
Hb concentration after (g/l)	84	176	150	202	159	157	132	194	10	<0.001	0.002	0.941	0.339	0.215	0.002
TPP concentration before (g/l)	84	61	54	70	157	59	49	74	4	<0.001	0.314	0.798	0.692	0.215	0.966
TPP concentration after (g/l)	79	65	58	76	149	63	52	80	4	<0.001	0.088	0.219	0.975	0.243	0.877
CK concentration before (U/l)	84	239	117	1633	159	237	105	1913	155	0.946	0.143	0.990	0.167	0.526	0.437
CK concentration after (U/l)	84	298	135	2008	159	295	103	2162	221	0.941	0.113	0.533	0.005	0.095	0.801
AST concentration before (U/l)	84	349	195	625	159	354	158	894	96	0.686	0.890	0.614	0.188	0.001	0.942
AST concentration after (U/l)	84	383	229	643	159	378	124	928	102	0.739	0.838	0.860	0.154	0.005	0.929
Lac concentration after (mmol/l)	84	13.1	2.1	31.3	159	18.6	5.9	34.4	5.0	<0.001	0.200	<0.001	0.385	<0.001	0.005
HR_av_ during BEFT (b.p.m.)^3^	41	178	138	204	56	189	166	210	11	<0.001	0.241	0.163	0.612	0.001	0.277
HR_peak_ in BEFT (b.p.m.)^4^	41	225	195	238	56	224	203	237	8	0.773	0.073	0.108	0.915	0.185	0.568
HR_min_ in BEFT (b.p.m.)^5^	41	115	95	142	56	125	81	154	13	0.001	0.288	0.791	0.463	0.001	0.451
HR at end of BEFT (b.p.m.)	41	194	123	229	56	203	136	229	21	0.067	0.152	0.608	0.248	0.022	0.178
HR 5 min recovery (b.p.m.)^6^	62	108	69	137	114	115	87	136	9	0.001	0.687	0.003	0.079	0.001	0.312
HR 5th min of recovery (b.p.m.)^7^	73	90	49	124	142	96	74	119	9	<0.001	0.745	0.002	0.060	0.001	0.162

RR=respiratory rate; RT=rectal temperature; Hb=Haemoglobin; TPP=total plasma protein; CK=plasma creatine kinase; AST=plasma aspartate amino transferase; Lac=plasma lactate.

1 Values presented as least square means (mean) and root mean standard error (RMSE) and minimum (min) and maximum (max) values.

2 The ratio of rider BW (riding tack not included) to horse BW.

3 Average HR during BEFT.

4 Peak HR in BEFT.

5 Minimum HR in BEFT.

6 Average HR during the first 5 min of recovery after the BEFT.

7 Average HR during the 5th minute of recovery after the BEFT.

Mean recovery HR during the 5-min recording period was 113±10 b.p.m. (range: 69 to 137 b.p.m., *n*=187) and HR during the last minute of the 5-min recovery period was 95±10 b.p.m. (range: 49 to 124 b.p.m., *n*=228). HR during the 5-min recovery period and HR during the last minute of that period were both affected by velocity (*P*<0.001) and both increased by 2 b.p.m. per km/h. Horses with scores ⩾5.5 for pace had higher recovery HR during the 5-min recovery period (114±1 *v*. 109±1 b.p.m., *P*<0.01) and during the last minute of the recovery period (95±1 *v*. 91±1, *P*<0.01).

Lac after the BEFT was 18.0±6.5 mmol/l (range: 2.1 to 34.4 mmol/l, *n*=266) and 72% of the horses had Lac ⩾15.0 mmol/l (range: 15.2 to 34.4 mmol/l), 15% had 10.0 to 14.9 mmol/l, 11% had 4.1 to 9.9 mmol/l and 2% had ⩽4 mmol/l. Velocity had an effect on Lac (*P*<0.001), with faster horses having higher values (1.3 mmol/l increase per km/h). The Lac after BEFT increased (*P*<0.01) by 0.4 mmol/l for every 1% increase in BWR. Horses awarded a score ⩾5.5 for pace had higher Lac (17.7±0.6 *v*. 14.0±0.6 mmol/l, *P*<0.001) than horses that received no score (=5.0) for pace.

The RR after the BEFT was affected by velocity (Table 3) and increased by five breaths/min per km/h. RT was affected by distance (Table 3) and was 0.2°C higher per km covered in the BEFT. CK was affected by distance (*P*<0.01) and was 114 U/l lower per km covered. Hb values after the BEFT increased by 0.8 g/l per 1% increase in BWR.

### Effect of sex

Stallions had lower RT than mares after the BEFT (*P*<0.05) and higher Htc, Hb and TPP both before and after the BEFT (*P*<0.001; Table 3). Stallions had lower mean and minimum HR during the BEFT, lower recovery HR during the 5-min recovery period and lower HR during the last minute of the 5-min recovery period than mares (*P*<0.001; Table 3). Stallions also had lower Lac than mares after the BEFT (*P*<0.001; Table 3).

### Effects of age group

Four-year-old horses had higher RR than 5-, 6- and ⩾7-year-old horses (117 *v*. 102, 97, 96 breaths/min, RMSE 28, *P*<0.05) after the BEFT. The 4-year-old horses had lower Htc (45% *v*. 46%, 46%, 47%, RMSE 2, *P*<0.05) and Hb values (161 *v*. 166, 168, 170 g/l, RMSE 10, *P*<0.01) than 5-, 6- and⩾7-year-old horses after the BEFT. The 5-year-old horses had lower Htc (46% *v*. 47%, RMSE 2, *P*<0.01) and Hb values (166 *v*. 170 g/l, RMSE 10, *P*<0.05) than the ⩾7-year-old horses after the BEFT.

### Judgement scores

Stallions received higher scores than mares (7.76 *v*. 7.57, RMSE=0.27, *P*<0.001) and 4-year-old horses received lower scores than the older age groups (7.48 *v*. 7.68, 7.75, 7.74, RMSE=0.27, *P*<0.01).

## Discussion

### Intensity of the BEFT

The physiological responses observed in our study confirmed that the BEFT is a high-intensity exercise. The high peak, average, average/peak HR and a high proportion of exercise performed at HR ⩾200 b.p.m., as well as marked increases in RR, RT, Htc, Hb, TPP, plasma CK, AST and Lac, show that the work load was strenuous and that anaerobic energy metabolism was necessary. However, there was considerable individual variation in the physiological responses to the BEFT.

It can be speculated that the peak HR observed was the maximal HR, as it was comparable to the maximal HR observed in racing Thoroughbreds (range: 204 to 241 b.p.m.; Krzywanek *et al.*, 1970) and Standardbreds (range: 210 to 238 b.p.m.; Åsheim *et al.*, 1970). The average HR (184 b.p.m.) during the BEFT was probably close to the lactate threshold (Persson, 1983) and, together with the amount of time spent at HR⩾200 b.p.m. (~3½ min), explains why the horses had high Lac. The Lac after the BEFT (18.0±6.5 mmol/l) was similar to that observed in Standardbred horses after a race (15 to 43 mmol/l; Ronéus *et al.*, 1999), in ‘high goal’ polo ponies after a training match (18.7±5.4 mmol/l; Ferraz *et al.*, 2010) and in eventing horses after an advanced 3-day event (19.1±4.2 mmol/l; White *et al.*, 1995). The variation in Lac in the present study could be explained mostly (36%) by sex, age group, distance, velocity, BWR and pace, whereas rider accounted for 14% of the random variation. However, the variation might also be explained by differences in muscle fibre composition and fitness (Lovell and Rose, 1991), although our study did not assess those parameters. Unfortunately, we had limited information on the training background of the horses, but according to the riders most (62%) horses were quite well prepared (preparation score >7.0). However, the only physiological parameter correlated (r⩾0.2, *P*<0.05) to the riders’ opinion was minimum HR (*r*=−0.25, *P*<0.05), which indicates that the riders’ opinions were poorly reflected in the physiological responses. It is possible that the trainers did not have physical preparation in mind when answering the question about level of preparation, but rather preparation for showing gaits that score high according the judging scale (FEIF, 2002). However, few significant (*P*<0.05) correlations between rider opinion and scores were found and the significant ones were weak. For example, there was a correlation between rider opinion and score for gallop (*r*=0.21, *P*<0.001) and with form under rider (*r*=0.18, *P*<0.01).

### Effect of sex

There was a clear effect of sex in the response to the BEFT. Although stallions covered longer distances (warm-up plus BEFT) and performed at higher speed, they had lower HR and Lac. This indicates that aerobic fitness was higher in stallions than in mares, which is also supported by the higher Htc and Hb values. A higher Htc in Standardbred trotter stallions than in mares has been shown previously (Persson *et al.*, 1996) and a higher Hb concentration has been found in stallions than mares of other breeds (Persson, 1967; Čebulj-Kadunc *et al.*, 2002). A significant sex difference in the proportion of type IIB fibres in the gluteal muscle has been found in untrained 4 to 5-year-old Icelandic horses, with a higher proportion in mares than in geldings, 53.5% *v*. 44.4% (Henckel *et al.*, 1994). In addition, all fibre types in geldings were surrounded by more capillaries than those found in mares (Henckel *et al*., 1994). A sex differences in fibre composition is also supported by findings in Thoroughbred and Standardbred horses, with stallions having a higher type IIA/IIB ratio in *m. gluteus medius* than mares (Ronéus *et al.*, 1991; Ronéus, 1993). Horses with higher oxidative capacity usually have higher type IIA/IIB ratio (Ronéus *et al.*, 1992) and, taken together, this indicates that male Icelandic horses have higher oxidative capacity than mares. Our results confirm findings by Persson and Ullberg (1974) in Standardbred horses, where stallions were suggested to have higher aerobic capacity than mares and geldings and findings on Thoroughbred and Standardbred horses, where males are reported to be faster than females (Árnason, 2001; Mukai *et al.*, 2003) and to have lower HR than females (Mukai *et al.*, 2003). The difference in aerobic fitness observed between stallions and mares in the present study might of course also be owing to differences in training practices, but according to the riders stallions were not better prepared than mares (7.3 *v*. 7.2, RMSE 1.5 and *P*=0.66).

In human athletes, it is commonly accepted that there are gender differences such as greater muscle mass, muscle strength and aerobic capacity in males that contribute to superior performance (Lewis *et al.*, 1986). Another reason for the superior performance observed in stallions in the present study might be lower body fat content compared with mares, as indicated by the lower BCS. In humans, it is known that body fat content is inversely related to both sprint and endurance running performance (Lewis *et al*.,^1986^) and similar observations have been made in horses (Kearns *et al*., 2002). There is no obvious explanation for the higher TPP observed in stallions, but they could have been more excited than mares and increased sympathetic activity can increase hydrostatic pressure and sweat losses, both of which could elevate TPP. Increased sympathetic activity in the stallions is also supported by their higher Htc before exercise. Training can also increase TPP (Fazio *et al.*, 2011), but as earlier mentioned stallions were, according to the riders, not better prepared than mares.

### Effects of age group

The 4-year-old horses had lower Htc and Hb values and higher post-exercise RR than the older horses, although they were ridden a shorter distance, for a shorter duration and at lower peak velocity (*P*<0.1). This indicates less aerobic capacity and might be a reason for the riders consciously spared their performance. The lower Htc values are in accordance with previous findings on Standardbred horses in training, where the Htc has been shown to increase up to 4 and 5 years in mares and stallions, respectively (Persson *et al.*, 1996). In the present study, Hb and Htc increased numerically up to ⩾7 years of age, indicating that fitness was improved. However, if an increase in age is associated with a more experienced and better-trained horse, more signs of improved fitness could be expected (e.g. reduced HR). The lack of such signs might indicate that the general level of fitness in this population was not improved much after 5 years of age. However, it is also likely that there were more horses in the older age groups that were less talented and might have spent more energy on showing good gaits.

### Effect of velocity and distance

As could be expected, velocity during the BEFT had a significant effect on several physiological parameters. It affected post-exercise Lac, exercise and post-exercise HR, and post-exercise RR, all of which increased with increased velocity. The higher the velocity, the more fibre types recruited and recruitment of type II fibres, particularly IIB, results in lactate production (Snow and Valberg, 1994). The effect of velocity on AST both before and after exercise could be related to common training practices and higher fitness in faster horses (Fazio *et al.*, 2011).

The distance covered seemed only to affect post-exercise RT and CK concentration. Similarly, Hodgson *et al.* (1993) showed a linear increase in RT with time and workload. The plasma activity of CK can reflect cell membrane permeability, which could be fatigue dependent (Anderson, 1975). Interestingly, the concentration of CK was lower as the horses were ridden a longer distance, although duration of exercise has been suggested to be an even more important factor than intensity for the release of CK during exercise (Anderson, 1975). Possible explanations could be that fitter horses were ridden longer and/or that horses with higher CK concentrations were not ridden longer because the riders perceived these horses to be already tired. It has been suggested that the higher response of glycolytic metabolism might be reflected in higher values of CK and AST because of permeability changes in muscle fibre membranes (Fazio *et al.*, 2011). CK and AST levels may also be affected by concentrate intake (Ribeiro *et al*., 2004) and transportation time (Leadon *et al*., 1989); however, in the present study, no correlations were found between these enzymes and diet and transport time (data not shown).

The distance covered during a BEFT should be 1.8 to 3.0 km (6 to 10 times of maximum 300 m each). In the present study, only 20% of horses were ridden <2.5 km and 41% were ridden ⩾3.0 km, which indicates that many riders do not comply with the BEFT instructions.

### Effect of pace

Flying pace is a high-speed gait in the BEFT (velocity can reach around 30 to 40 km/h) and horses showing pace not only covered a longer distance but also at a higher average speed. Pace is also a gait likely to increase energy expenditure compared with the most economical gait at this velocity gallop. It has been found that ponies appear to choose a gait that maximises energy efficiency and that if they are forced to use extended gaits the VO_2_ can increase by up to 70% (Hoyt and Taylor, 1981). Altogether this may explain why horses that showed the pace gait had significantly higher Lac and recovery HR than horses not showing pace.

### Scores

We believe that the horses participating in this study were representative of the population of Icelandic horses attending a BEFT, as there was no difference in total score for riding abilities between horses participating in the study compared with non-study horses. According to Árnason (1984, Porvaldur Árnason personal communication, 2013), age is important for scores and an increase in total score (conformation and riding abilities) can be expected yearly from 4 to 7 years old. In the present study, we compared the total score for riding abilities, for which 4-year-old horses had lower scores than older horses.

### Weather and correlations with physiological parameters

Because of the low ambient temperature, low humidity and windy conditions, heat loss through conduction, convection and evaporation was facilitated. It has long been known that air resistance increases energy expenditure during running and walking in humans (Pugh, 1971), and interestingly in the present study, HR increased as wind speed increased. Actually a few riders decided not to finish the BEFT (horses excluded from the study) because of the strong wind speed (the day with mean 12.9 m/s).

## Conclusions

This study showed that the riding assessment in a BEFT is a high-intensity exercise and maybe even, for some horses, supramaximal exercise. The study also showed that aerobic fitness was higher in stallions and that age had a limited effect on the physiological response. These results should be used as a guide for the development of training programmes and fitness tests in Icelandic horses that would improve both performance and welfare of the horses.
